# The Occurrence of Skeletons of Silicoflagellata and Other Siliceous Bioparticles in Floral Honeys

**DOI:** 10.3390/foods10020421

**Published:** 2021-02-14

**Authors:** Donát Magyar, Paulian Dumitrica, Anna Mura-Mészáros, Zsófia Medzihradszky, Ádám Leelőssy, Simona Saint Martin

**Affiliations:** 1 National Public Health Center, 1097 Budapest, Hungary; 2 Institute of Earth Sciences, Université de Lausanne, 1015 Lausanne, Switzerland; Paulian.Dumitrica@unil.ch; 3 Faculty of Biological Sciences, Friedrich Schiller University Jena, 07743 Jena, Germany; muram.anna@gmail.com; 4 Museum and Library of Hungarian Agriculture, 1146 Budapest, Hungary; medzihradszky.zsofia@mmgm.hu; 5 Department of Meteorology, Eötvös Loránd University, 1053 Budapest, Hungary; leelossyadam@gmail.com; 6 Centre de Recherche en Paléontologie, Muséum National d’Histoire Naturelle, Sorbonne Université, 75006 Paris, France; simona.saint-martin@mnhn.fr

**Keywords:** honey, Silicoflagellata, diatoms, pollen, spores

## Abstract

Siliceous marine microfossils were unexpectedly discovered during the analysis of flower honey samples from Poland and Tunisia. The microfossils were represented by protist with siliceous skeletons: silicoflagellates, diatoms, and endoskeletal dinoflagellates. This is the first record of such microfossils in honeys. Based on the high percent of anemophilous pollen grains and spores in the sample, it was hypothesized that silicoflagellates were deposited from the air onto the nectariferous flowers, then bees harvested them with the nectar. Based on the comparison of pollen content of honeys and flowering calendar of Tunisia, the harvest time of honey was identified as a period between 1 April and 31 May 2011. Trajectory analysis of air masses in this period confirmed that siliceous microfossils could be aerosolized by wind from the rocks of the so-called Tripoli Formation of Messinian age (6–7 Ma). Similar to the Tunisian case, the Polish trajectory simulation also supports the hypothesis of atmospheric transport of silicoflagellates from outcrops of Oligocene age in the Polish Outer Carpathians. In the case of diatom content of honey, however, the source can be both natural (wind) and artificial (diatomaceous earth filters). For a correct determination, natural sources of siliceous bioparticles, such as wind transport from nearby outcrops should be also considered. Silicoflagellates could be used as complementary indicators of the geographical origin of honeys collected in areas characterized by diatomite outcrops, supporting the results obtained with other methods; thus, such indicators merit further studies within the area of honey authenticity.

## 1. Introduction

The identification of the origin of food is one of the most important issues in food quality control [1]. Depending on its geographical origin (the region where the beehives are located and the surrounding environment), honey can acquire different characteristics and properties. Therefore, geographical origin is an important parameter with respect to honey differentiation and valorization.

The determination of the geographical origin of honey relies on microscopical examination of its pollen profile if it is specific enough in the area of interest. Because of the limitations of this method (being expensive, time-consuming, and strongly dependent on the qualifications and judgement of the analyst), there is a tendency to replace pollen analysis by finding other markers for honey discrimination. Minerals and trace elements [1], and fungal spore content [2] are some of the parameters that have been examined for the recognition of the origin of honeys. However, when microscopical analysis of honeys is performed, one can see a great variety of particles, other than pollen grains or spores. Some of them belong to insect parts, most commonly bee hair, tracheae and, especially in *Pinus* honeydew honeys, wax produced by Pseudococcidae. Surprisingly, some particles come from microfauna as well (Acari, Rotifera, eggs of Tardigrade). Plant trichomes, starch, and phytoliths are also common components of honey samples. As a sign of human activity, microplastics can also be observed in honey under the microscope. These particles are often overlooked during the routine melissopalyonolgical analysis of samples. As no attention is paid to them, they remain unidentified, although they would be useful in the analysis.

During our research to find new indicators of the origin of the honeys, unusual multiradiate structures were found in honey samples that originated from Poland and Tunisia. Therefore, we aimed to identify these particles and other accompanying components in honey to find any indication of their origin.

## 2. Materials and Methods

The honeys were purchased from food shops in Poland (according to the information on the product label, the honey was harvested from *Fagopyrum esculentum* in Stróze, near Nowy Sącz, 2013) and Tunisia (mixed floral honey, Nabeul, 2011). Ten grams were taken from 250 g of previously homogenized honey, dissolved in 20 mL of distilled water at 40 °C, centrifuged for 5 min at 2500 rpm, and allowed to settle. The sediment was recovered in 10 mL of distilled water and again centrifuged. The sediment was then collected with a Pasteur pipette and dried onto microscope slides at 40 °C. It was then mounted in glycerine-gelatine and covered [3,4]. The entire surface of the preparation was scanned under 600× magnification of an Olympus CX 31 microscope. Preliminary identification indicated that multiradiate particles may belong to extinct microscopic organisms, occurring as fossils.

To see the frequency of occurrence of these particles, we studied samples from a collection containing 106 honeys, prepared according to the method mentioned above. These samples were listed in the Supplement 1 with their collection code, type, botanical origin, and location.

Because the Polish (P) and the Tunisian (T) honeys were particularly rich in the investigated multiradiate particles, further analyses were performed. To identify the geographical origin of the multiradiate particles, a combination of methods was applied. First, fungal spore and pollen composition were determined.

**Figure 1:**
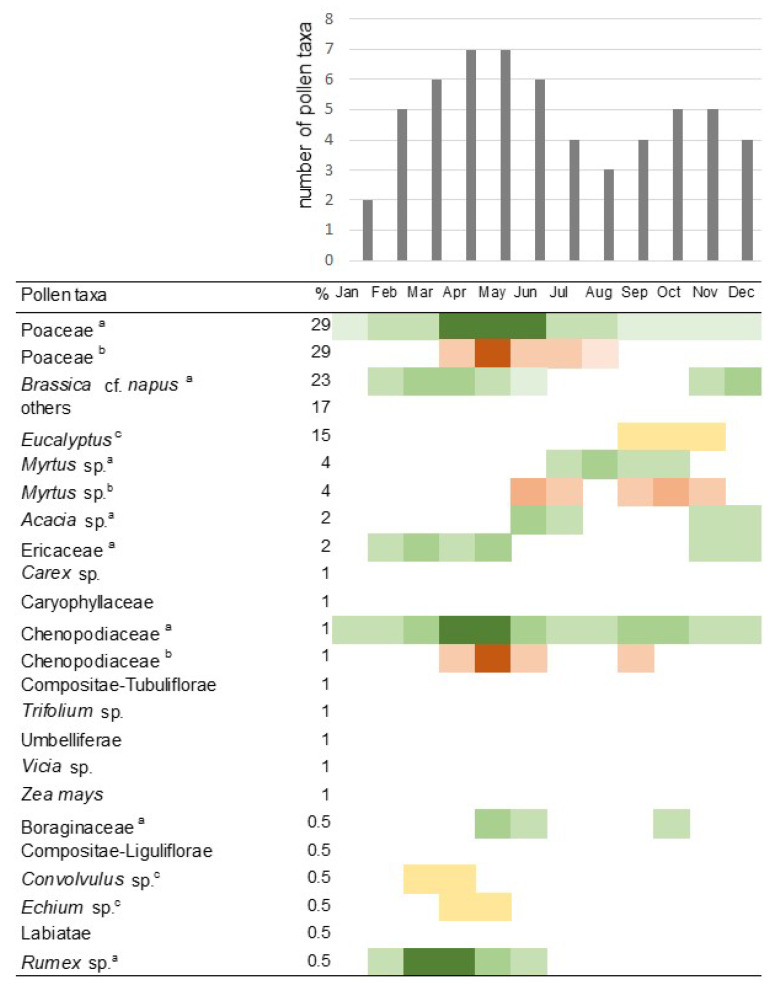
Combined aerobiological and phenological calendar of pollen taxa found in Tunisian honey. ^a^: Flowering time from aerobiological data [8] covering northern Tunisia shown in green; deep green indicates the main pollination period. ^b^: Flowering time from another aerobiological dataset [8] covering Tunisia shown in red. Dark shades mean flowering peaks, according to the original illustrations. ^c^: Flowering time from phenological data [9] shown in yellow. ‘Pollen taxa %’ means the relative abundance of pollen taxa in the Tunisian honey. In [8], taxa are referred to as: Amaranthaceae (instead of Chenopodiaceae), *Erica* (instead of Ericaceae), *Borago* (instead of Boraginaceae), Brassicaceae (instead of *Brassica* cf. *napus*), and Myrtaceae (instead of *Myrtus*), in [10]: ‘Graminees’ (instead of Poaceae), ‘Myrtacees’ instead of *Myrtus*), in [9]: *Eucalyptus gomphocephalla* (instead of *Eucalyptus*). Polygalaceae, found in low numbers (<0.5%) are not shown, as flowering data is not available for this region.

Among fungal spores, honeydew indicators were not found [2], but indicators of floral origin (*Metschnikowia reukaufii,* P,T) and common airborne fungi were present (*Alternaria* sp. P,T, *Aspergillus/Penicillium* P,T, *Bipolaris spicifera* T, *Botrytis* sp. P, *Chaetomium* sp. P,T, *Cladosporium* spp. P,T, *Coprinus* sp. P,T, *Curvularia* sp. T, *Diplodia frumenti* T, *Drechslera biseptata* T, *Drechslera/Helminthosporium* T, *Ellisembia* sp. T, *Epicoccum nigrum* P,T, *Ganoderma* sp. P, *Leptosphaeria* spp. P, *Melampsoridium* sp. T, *Paraphaeosphaeria michotii* P, *Periconia* sp. T, Peronosporaceae P, *Pithomyces chartarum* T, *Polythrincium trifolii* P, Pucciniaceae T, *Rhizopus* sp. T, *Stemphylium* sp. P,T, Telephoraceae P, *Torula* sp. P,T, *Trichothecium roseum* P, *Tripospermum* spp.P, Ustilaginomycetes P,T, other Ascomycota). Pollen content was expressed as percentage of pollen grains (N = 300) [5]: P: *Brassica* 34%, *Centaurea cyanus* 5%, *Fagopyrum* 5%, *Trifolium* 2%, Ericaceae and *Tilia* < 2%; T (in descending order of frequency; data are shown on Figure 1): Poaceae spp., Brassicaceae (*Brassica* cf. *napus*), *Eucalyptus* sp., *Myrtus* sp., *Acacia* sp., Ericaceae sp., *Carex* sp., Caryophyllaceae, Chenopodiaceae, Compositae-Tubuliflorae, *Trifolium* sp., Umbelliferae, *Vicia* sp., *Zea mays*, Boraginaceae, Compositae-Liguliflorae, *Convolvulus* sp., *Echium* sp., Labiatae, Polygalaceae sp., *Rumex* sp. [6,7]. Percentage of pollen grains also showed that dominant taxa are anemophylic.

Consequently, we hypothesized that multiradiate particles found in the honey samples might also have airborne origin. To test this hypothesis, we searched for the possible source of multiradiate particles. The source of airborne particles can be identified with the calculation of wind trajectory of air masses carrying particles from long distances. An important information for calculations is the time (year and month) of honey harvest. However, in case of the Polish honey, only the year (2013) was known. Harvesting period was determined to be July–August according to the blooming of *Fagopyrum esculentum* [11]. In the case of Tunisian honey, the year 2011 was shown on the product’s label. The month of acquisition was known as well (31 July). Therefore, the month of harvest had to be identified. With this aim, a forensic palynological method was used [12,13]. To collect information on pollination of local melliferous plants, phenological calendars were reviewed [9]. Because the dominant pollen taxa in the honey belonged to anemophilous plants, data from aerobiological literature were also considered [8] (data from the year of 2011) [10]. Number of pollen taxa found in the honey was summarized by month and illustrated on a histogram (Figure 1). According to this analysis, the honey was most probably harvested during the months of April and May.

To investigate whether atmospheric conditions supported the transport of aerosol particles from the suspected source area to the harvesting region, an atmospheric dispersion model was applied for the flowering periods (P: July–August 2013, T: April–May 2011). The dust emission flux was estimated to be a cubic function of the friction velocity, according to the dust emission model presented by Bagnold [14], discussed more recently by Xuan [15], and applied as described in a previous study [16]. The threshold friction velocity was set to 0.5 ms^−1^, a medium value within the range of experimental results of Marticorena and Bergametti [17]. If the friction velocity was higher than the threshold friction velocity and no precipitation occurred, 1000 particles were released in every hour from each of 20 levels between 1–1000 m above ground; and their atmospheric trajectories were simulated for 48 h. Meteorological data was obtained from the GDAS FNL (Global Data Assimilation System—Final Analysis) database [18] with 3 h temporal and 0.25° spatial resolution. Atmospheric dispersion was simulated with the Lagrangian particle dispersion model RAPTOR that calculates advection, turbulent dispersion, and deposition [19,20]. As the extent and amount of mobilizable dust is unknown, sensitivity maps were produced with unit m^−3^, normalized to a total sensitivity of 1 over the entire domain. This way, the spatial and temporal pattern of the dispersion could be investigated while the amount of deflated dust remains unknown.

## 3. Results and Discussion

**Figure 2:**
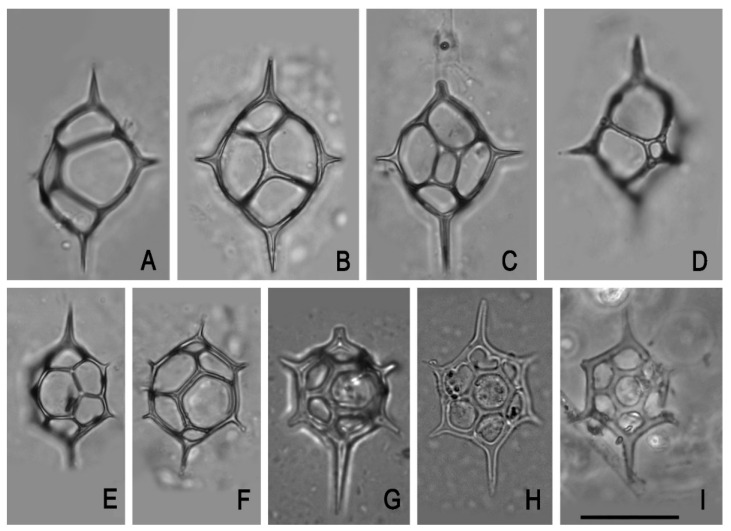
Silicoflagellates and diatoms found in floral honey. (**A**,**B**). *Dictyocha fibula*. (**C**,**D**). *Distephanopsis crux*. (**E**). *Stephanocha speculum speculum*. (**F**)**.** *Stephanocha speculum speculum* f. *notabilis*. (**G**–**I**). *Stephanocha speculum.* (**A**–**F**) from Tunisia, (**G**) Poland, (**H**) Romania, (**I**) Greece. Scale bar: 20 µm.

**Figure 3:**
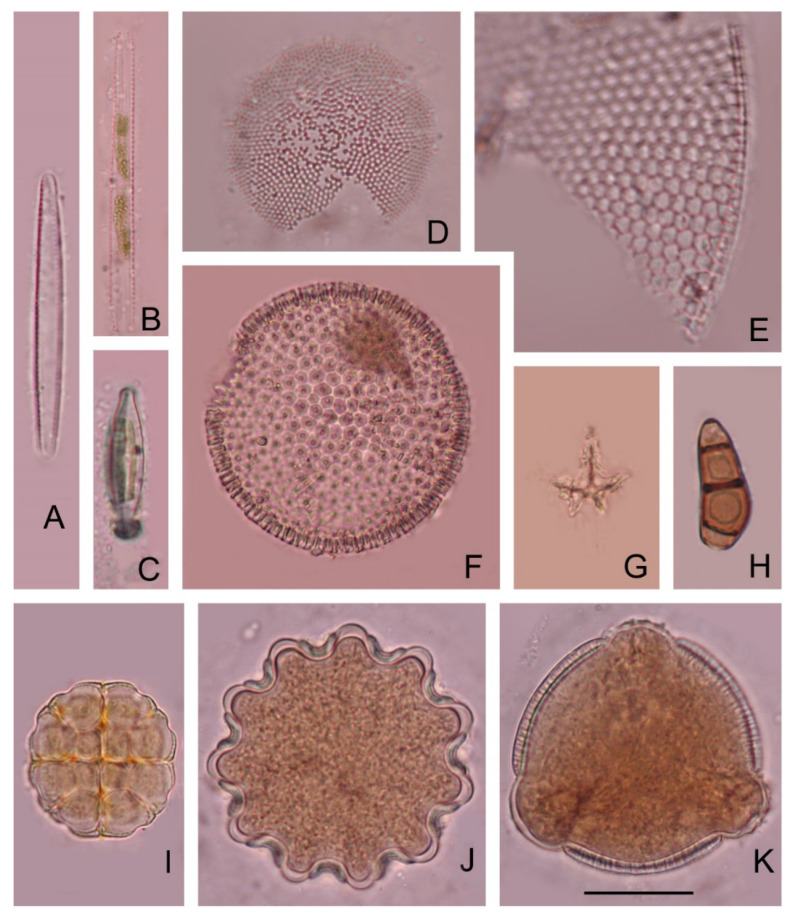
Diatoms, dinoflagellates, fungal spores, and pollen grains found in floral honey from Tunisia. (**A**,**B**). *Thalassionema nitzschioides.* (**C**). *Mastogloia* sp., (**D**). *Actinocyclus divisus.* (**E**). fragment of *Coscinodiscus*. (**F**). *Coscinodiscus marginatus,* (**G**). *Actiniscus pentasterias.* (**H**). *Curvularia* sp., (**I**). *Acacia* sp., (**J**). Polygalaceae sp., (**K**). *Convolvulus* sp., Scale bar: 20 µm.

Our investigations have shown that honey from Tunisia (T), Morocco (M), Africa (A), Greece (G) Poland (P), and Romania (R) contained silica skeletons of planktonic marine Silicoflagellata belonging to *Dictyocha fibula* (T), *Distephanopsis crux* (T), *Stephanocha speculum* (A,G,P,R), *Stephanocha* cf. *speculum* (M), *Stephanocha speculum speculum* (T), *Stephanocha speculum speculum* f. *notabilis* (T) (Figure 2, Table 1). Diatoms, e.g., *Actinocyclus divisus*, *Coscinodiscus marginatus*, *Coscinodiscus* (?) sp., *Fragilaria* (?) sp., *Hantzschia amphioxys*, *Mastogloia* (?) sp., *Melosira* sp., *Nitzschia* (?) sp., *Thalassionema nitzschioides*, and very rare endoskeletal siliceous dinoflagellates belonging to *Actiniscus pentasterias* were also found (Figure 3, Table 1). To our knowledge, this is the first record in the literature of silicoflagellates and other protists with siliceous skeletons occurring in honey.

Silicoflagellates are planktonic marine chloroplast-bearing protists with a flagellum and a siliceous skeleton formed of distally closed hollow bars known to have existed starting from the mid-Cretaceous (Albian) to recent. Their skeletons usually comprise 1–2% of the siliceous component of marine sediments [21] and in some cases, as for example in some Sarmatian deposits from Romania, they are so abundant that practically these rocks could be called silicoflagellitites. Their skeleton has a rather simple geometrical form and consists usually of two parts: a basal ring and an apical structure, both interconnected by bars. All these elements have a special descriptive nomenclature [21,22]. *Dictyocha fibula* is a species characteristic of warm water, whereas *Stephanocha speculum* is much more frequent in colder waters. *Distephanopsis crux* is a Miocene and Pliocene species that became extinct at the base of the Pleistocene [23]. All these three species are common in the diatomites of the so-called Tripoli Formation of Messinian age that can be visible in outcrops and found also in cored sediments [24,25,26,27] in the Mediterranean area. They were deposited 6–7 million years ago before the period of the closing of the Mediterranean Sea, which determined the famous “Messinian Salinity Crisis” that lasted until the Pliocene [28]. These diatomites are well known especially in Spain, Italy, Crete, Cyprus, and in the northwestern part of Africa, in Morocco and Algeria. The only mining of Messinian diatomites, formerly active in the Oran region in Algeria [29], has long been abandoned.

The diatomites are porous rocks of marine or lacustrine origin. The marine diatomites originated in zones of high planktonic fertility. The lacustrine diatomites were especially formed in lakes of volcanic craters or in zones with volcanic tufs. Although they are very light due to the porosity of diatom frustules, they may contain up to 3000 frustules/mm^3^ [30]. Therefore, we hypothesized that these marine siliceous microfossils found in honey are of airborne origin. They may have been deposited as airborne dust on the flowers from where the bees collected them with the nectar. The presence of other particles (pollen grains, fungal spores, dinoflagellates, and diatoms) corroborate this hypothesis.

Diatoms can become airborne from outcrops (e.g., by mining activity), by deflation from dried lakebeds, or after swamp fires and storms [31] or via sea-foam and bursting bubbles [32]. Geissler and Gerloff [33] showed that the species composition of airborne diatoms above the city of Berlin is identical to the composition of diatoms in Berlin lakes and rivers. Folger [34] as well as many others, found *Melosira granulata* and *Stephanodiscus astrea* to be the most common diatoms in airborne dust samples from the Equatorial Atlantic. *Cyclotella* and *Stephanodiscus* spp. were found in high quantities as house dust in a building constructed on a dried lakebed in Hungary (Magyar, unpublished observation). Specimens of *Corethron*, another marine diatom, were recently detected in Late Cretaceous amber [35]; the authors considered that the amber forest grew in a nearshore environment where wind introduced the marine diatoms into the terrestrial realm. The occurrence of airborne algae in the atmosphere has been recorded as early as the middle of the 19th century. In 1833, aboard the famous vessel Beagle, Charles Darwin observed airborne diatoms in the dust from North Africa deposited on the board when it was near Cape Verde Islands [36]. Ehrenberg [37] reported 18 species of freshwater diatoms from the dust samples sent by Darwin. Since then, North African dust particles associated with diatoms were frequently observed [38]. Diatoms from the Bodélé Depression (once part of Mega-Lake Chad, North Africa) are the main source material for the dust [39]. Direct sampling of the atmosphere in various environments (e.g., terrestrial, marine, and freshwater) provided evidence that airborne algae are naturally occurring in the aerial biota [40]. In the Tunisian honey we investigated, recent diatoms were present. *Mastogloia* sp. and *Thalassionema nitzschioides* contained chloroplasts, thus they could be recent, originating from sea spray or high tide and wind.

*Actiniscus pentasterias* is a Miocene to recent species of endoskeletal dinoflagellates. Its star-like specimens with five, rarely four arms are frequent in marine sediments with other siliceous microfossils [41]. Its stellate arched structure, although rather small, is very easily recognized in microscope slides. Each spicule represents one of the two spicules present in a living cell and disposed symmetrically face to face with their concave sides opposed in the ovoid cell. Usually, they are separated in fossil material, but interconnected specimens by the end of the rays can be encountered when the sample is not treated too much with hydrochloric or other acids that can dissolve the points of interconnection. Rather neglected in the fossil samples, it was studied in detail by Dumitrica [41] who described several species and tried to make an order in this group.

There are no diatomites and therefore no mining of diatomites in Tunisia. However, the closest Messinian diatomites are those of Sicily about 250 km to 300 km from Nabeul (the location of honey harvest) to the northeast [26,27].

**Figure 4:**
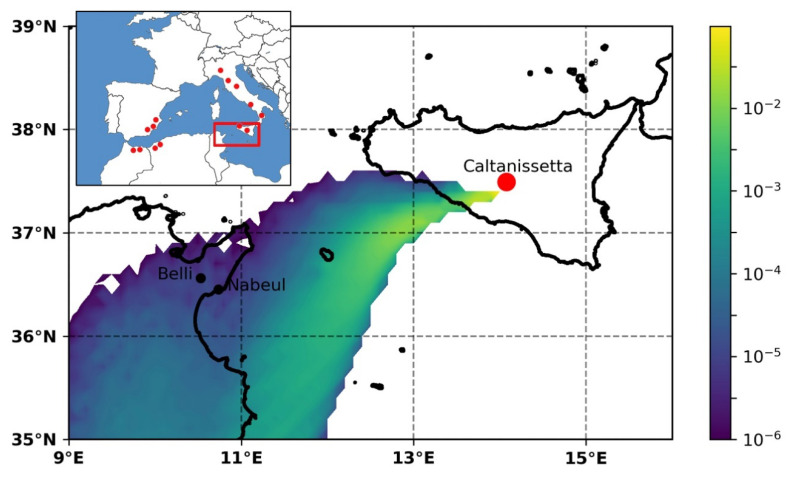
An episode of air mass possibly transporting microfossils from the emission area (Caltanissetta, Sicily) to the honey harvesting area shown on an atmospheric dispersion sensitivity map [m^−3^] for the dust deflated between 18–6 UTC, 21–22 May 2011. Red dots in the small map show the distribution of Messinian diatomites.

Atmospheric dispersion simulations performed in the period April–May 2011 revealed a situation when atmospheric conditions supported the transport of dust from Sicily to the region of Nabeul. Dust emission was assumed only when the threshold friction velocities exceeded 0.5 ms^−1^ and no precipitation occurred. Among these, 21–22 May was characterized by northerly-northeasterly winds in Sicily, brought by a Mediterranean cyclone marking the end of an 18-day long drought. Dry surface conditions with approaching thunderstorms were ideal for gust fronts and evaporative cooling, a well-known pattern for deflation [42]. According to WMO synop reports from the hilltop meteorological station of Enna (20 km to the northeast and 350 m above Caltanissetta) on the night of 21–22 May 2011, repeated thunderstorms with or without precipitation occurred, although yielding a total precipitation of only 3 mm/12 h. This confirms the potential for evaporative cooling and the formation of gust fronts. Thunder with no rain and the 1-h mean wind velocity reaching 30 km/h was reported at 3 UTC. Continuous rain inhibiting further deflation initiated at approximately 6 UTC; however, the total precipitation remained relatively low during the day (12 mm/24 h). Atmospheric dispersion maps of particles released from Caltanissetta between 18–6 UTC on 21–22 May 2011 (Figure 4) confirm the potential of the deflated dust to reach Tunisia. In the flow of the cyclone, the dust would have travelled in a moist environment to North Africa and deposited efficiently with rain onto the surface.

It might also be noted that in the previous week (10–18 May 2011), a documented Saharan dust event had occurred in Portugal [43], related to the ongoing shallow cyclonic activity over the Mediterranean. While dust transport typically occurs northward on the leading edge of cyclones, similar dust transport potential is present on the rear edge of a cyclone towards North Africa.

Therefore, trajectory simulations support the hypothesis of atmospheric transport of silicoflagellates from Sicily to Nabeul.

**Figure 5:**
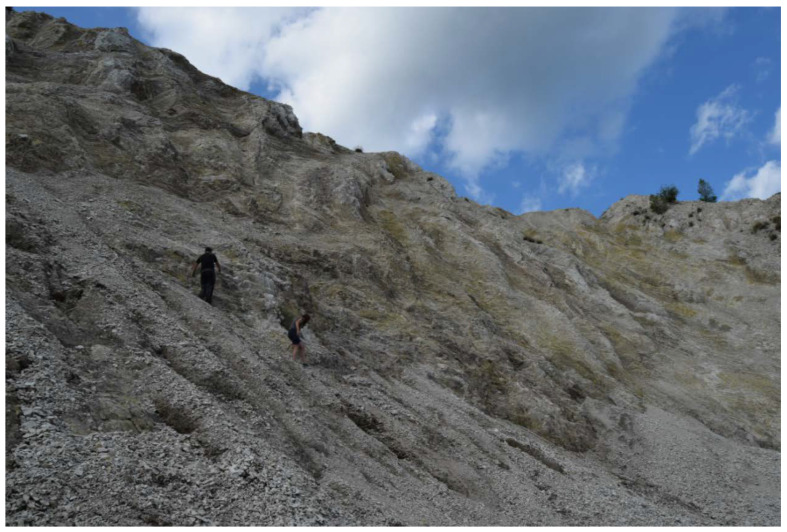
A quarry located in the Carpathians, Sibiciu de Sus, Romania—a possible source of airborne microfossils. Photo courtesy of Emilia Tulan.

**Figure 6:**
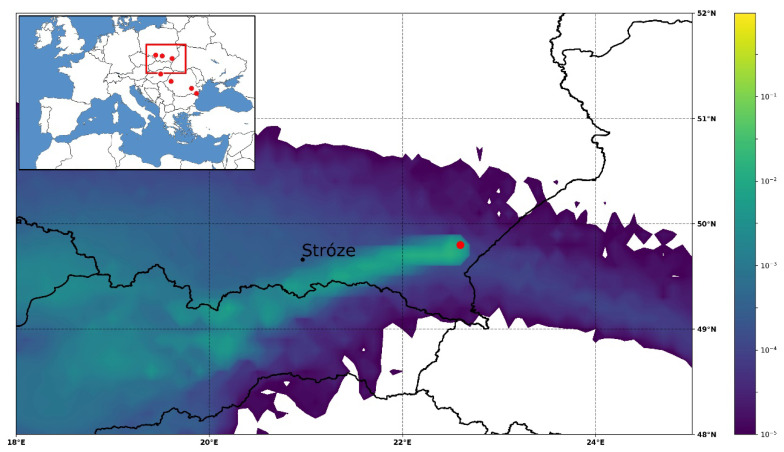
An episode of air mass possibly transporting microfossils from the emission area (red dot) to the honey harvesting area near Stróze, Poland; shown on an atmospheric dispersion sensitivity map [m^−3^] for the dust deflated on 26 August 2013. Red dots on the small map show the distribution of Oligocene-Miocene diatomites.

*Stephanocha speculum* was found in a Polish product labelled as *Fagopyrum* honey. Since this silicoflagellata occurs from the Miocene to Recent, one can suppose that it comes from the diatomite intercalated in the Middle Miocene from the Silezian Basin [44]. Outcrops of Oligocene age diatomites in the Carpathians are exposed on the surface (Figure 5); thus, particles as small as silicoflagellate skeletons can be lifted from outcropping on the surface of soft sediments and transported by winds, being a plausible source of silicoflagellates to explain our observations. It is possible that the source of these silicoflagellata is a diatomite in eastern part of the Polish Outer Carpathians, 60 km from the honey harvesting area [45]. A simulation study was performed with the atmospheric dispersion model for the harvesting period July–August 2013, i.e., the blooming of *F. esculentum* [11]. The source area was represented by the location 49.8 N and 22.6 E and sensitivity maps were produced for each day to investigate whether the atmospheric conditions were suitable to deflate particles and transport them to the harvesting location near Stróze, Poland. It was found that in the beginning and the end of the harvesting period, e.g., on 4 July and 26 August, atmospheric conditions supported the potential transport of microfossils to the honey harvesting area (Figure 6). Meteorological observations reported from Nowy Sącz, located in a distance of 20 km from Stróze, confirmed that between 10–19 UTC on 4 July and 9–16 UTC on 26 August 2013, easterly-northeasterly winds dominated the area, potentially transporting microfossils from the upwind direction. No precipitation but trace had been reported for the previous four days. Similar to the Tunisian case, the Polish trajectory simulation also supports the hypothesis of atmospheric transport of silicoflagellates from outcrops of Oligocene age (possibly quarries).

The dominance of the Poaceae pollen in the Tunisian honey sample corroborates our hypothesis that the particles were deposited from the atmosphere. Poaceae produce typical anemophilous pollen grains, and their presence in honey is rare and incidental in North African honeys [46]. Fungal spores were also the common members of the airspora.

Surprisingly, we did not find any scientific report on the presence of silicoflagellata in air samples, possibly because aerobiological networks focus on the monitoring of pathogenic bioaerosols, e.g., allergenic pollen grains and spores, rather than other particles [47]. Further studies are needed to study the presence of silicoflagellates and diatoms in air samples and honeys harvested within the area of diatomite outcrops.

In our honey collection, a total of 21 countries are represented by samples, 14 of them from Europe (most of them were from Italy, Greece and Hungary). Silicoflagellates were observed in three of our European honey samples (*Pinus* honeydew honey from Greece; *Fagopyrum esculentum* honey from Poland, Stróze; and honeydew honey from Romania, Odorheiu Secuiesc). No silicoflagellates were found in eight other *Pinus* honeydew honey samples collected from Greece, and four *Fagopyrum esculentum* samples collected from Poland, Stróze or nearby (Królów, Lipowy and Wiśniowa). Other two samples (*Robinia pseudo-acacia* honey and a honeydew honey) collected from Stróze or nearby (Pogorzany) were analysed, but again, with negative results.

In case of North African honeys, samples from Egypt, Morocco, and Tunisia were available for analysis. Silicoflagellates were found in the honeys originating from the latter two countries. For Morocco, there is a possible source from Messinian diatomite outcrops known in Boudinar and Melilla basins and for Tunisia the closest possible source may be the Messinian diatomites outcrops in Sicily [48,49]. Another positive sample was found from Africa, but more precise information on the origin of the honey is not available. Because data on the composition of the North-African honeys are uncommon [9], information on new indicators of geographical origin of honeys in this region is useful. According to our findings, the occurrence of silicoflagellates may be expected in honey harvested near diatomites in Algeria, Crete, Hungary, Poland, Romania, Sicily, and Spain. We propose further studies on the presence of microfossils in honeys of other areas of the world as well.

The identification of the origin of food is one of the most important issues in food quality control [1,50]. Considering the increasing global trade and owing to the higher economic value of specific honeys (e. g. those having protected geographical indication), such products are targets of adulteration through incorrect labelling and fraudulent admixing with honey of lower value and quality. Thus, in order to promote fair competition among producers, and protect consumers, there is a growing need to assess the authenticity of honey, particularly with regard to geographical origins [1]. Microfossil identification could be compared with other microscopy-based analytical methods, such as melissopalynology. These methods allow a good differentiation of honeys, however, are not suitable for application in the case of filtered honeys. Melissopalynology has a limitation in its application in honey adulterated by pollen addition [1]. Silicoflagellates can be used as non-quantitative indicators, as the mere presence of their distinct siliceous skeletons can indicate the geographical source of honeys. Because no particle counting is needed, the analysis is less time-consuming than quantitative microscopical methods. (Similar, non-quantitative indicators of honey origin were previously proposed on the basis of biodiversity of fungal spores [2].) Our findings encourage the confirmation of honey origin also by recording the occurrence of microfossil elements during routine melissopalynological analysis. Silicoflagellata skeletons are characteristic multiradiate particles and it is easy to observe them in honey samples, thus they seem to be good candidates of indicators of geographical origin of honeys in food analysis. The most frequent species was *Stephanocha speculum* (1 particle/g honey), followed by *Dictyocha fibula* and *Distephanopsis crux* (both having 0.3/g; data from the Tunisian sample). Only *Stephanocha speculum* was found in the other honey samples from Africa and Europe. The limitation of our method is the low frequency of microfossils in honeys. Observation of the presence of silicoflagellates can be used in a complementary way. Because of their low frequency in honey, only positive findings can be interpreted as indicators of geographical origin. Apparently, silicoflagellates in honeys are not common, but if detected, can provide a strong evidence of geographical origin of the honey.

Marine species of diatoms (*Coscinodiscus marginatus*, *Actinocyclus divisus* and *Thalassionema nitzschioides*) have a large geological range and are also known in actual assemblages. They are known in Messinian diatomites in the Mediterreanean area, but since they have a very large geological time span distribution, they cannot be considered as markers for Messinian [26,48]. Thus, silicoflagellates only support a possible Messinian age. Consequently, diatoms cannot be used as indicators of the honey origin. It should be mentioned that fossil diatoms can have another source in the honeys: diatomite earth filters. The use of diatomite earth filters is largely known in food industry. The presence of fossil diatoms was already observed in honey, and is explained by the use of diatomite filters in order to: (a) filtration to obtain pure honey [1] (b) forge [51,52]. Forgers combine two kinds of honey: a local honey with another kind of honey that is much cheaper. Diatomaceous earth filter aids completely remove the pollen, and thus prevent any identification of source by analysis of pollen. In our samples, three categories of diatoms were found: fossil marine diatoms, freshwater diatoms, and living freshwater pennate diatoms.

1. Fossil marine diatoms (ex. *Coscinodiscus marginatus*, *Actinocyclus divissus*) together with fossil silicoflagellates. Their occurrence can be explained:

(a) By wind transport from fossil diatomite (e.g., Tripoli formation);

(b) Contamination from filters made from diatomite earth. Diatomite used for the fabrication of filters may be made from marine diatomites that contain marine diatom genera like *Coscinodiscus* and *Actinocyclus*.

2. Freshwater centric diatoms (e.g., *Aulacoseira distans*, *Cyclotella*), known from Miocene, Pliocene up to day in freshwater and lacustrine assemblages. Their occurrence might be explained:

(a) By wind transport from fossil diatomite from strata of fossil freshwater diatoms.

(b) Contamination from filters made from diatomite earth. For example, in France, there is known exploitation of fossil lacustrine diatomite Miocene in age from Massif Central that might be used for the fabrication of filters [53]. This lacustrine diatomite contains *Aulacoseira distans* and *Cyclotella*. Recovery of *Aulacoseira* sp. was reported from only pressed honey sample in Nigeria, Africa, and interpreted as an indication of secondary contamination during processing [46].

3. Living freshwater pennate diatoms that present the chloroplasts and cellular content, together with living cyanobacteria (*Chroococcus* and *Oscillatoria*) and green algae (*Scenedesmus*). Obviously, living diatoms do not originate from diatomite earth filters. Their occurrence might be explained:

(a) By wind transport. *Nitzschia* and *Chroococcus* were common living diatom and cyanobacteria in our samples (especially in Cuba, France, Greece, Hungary and Italy). These genera were reported to be airborne [54,55].

(b) The presence of green microalgae (e.g., *Scenedesmus*) is characteristic of honeydew honeys as well [56]. When honey bees collect honeydew, they may also collect other attached structures such as algae that grow on plants. In our samples, such algae were not associated with silicoflagellata, but were found in honeys of honeydew origin.

(c) Freshwater, as a source cannot be excluded either. Adding water to honey is a known authentication technique to increase the volume. Food analytical tests, such as microscopic yeast count and analysis of fermentation products are available to indirectly detect this type of honey adulteration [1].

Each of the above-mentioned hypotheses can be considered as true alone, but they are not exclusive (i.e., diatoms and silicoflagelates transported by wind and living diatoms were added using freshwater).

In case of the Tunisian honey, matching evidences (wind trajectory analysis and high percentage of anemophilous pollen and spores) indicate that the source of siliceous bioparticles is possibly not the result of filtration, but air. In the unknown African honey, a high percentage of anemophilous pollen (Chenopodiaceae) was found too, leading to similar hypothesis. The Polish product was labelled as *Fagopyrum* honey. Our pollen analysis confirmed the presence of this pollen. Here, again both trajectory analysis and pollen composition suggested natural sources of particles in the honey. In the Romanian honey, fungal indicators (*Metschnikowia*, *Retiarius* and *Tripospermum*) suggested that the origin is mixed floral and honeydew honey, but of natural (i.e non-forged) source [2]. Similar evidences are available in the honey sample from Altenst, Germany, where conidia of a honeydew-indicator hypomycete (*Retiarius*) were found with the freshwater diatom *Aulacoseira distans*. Consequently, the presence of siliceous bioparticles in honey does not necessarily indicate the manipulation of the product. For correct determination, natural sources of siliceous bioparticles, such as wind transport from nearby outcrops, should also be considered. According to a world map showing main directions of atmospheric transport of diatoms, presented by Harper and McKay [57], the occurrence of windborne microfossils in honeys might be a world-wide phenomenon.

## 4. Conclusions

Silica skeletons of planktonic marine silicoflagellates were found in honey samples from Greece, Morocco, Poland, Romania, and Tunisia. In Tunisia, the source of silicoflagellate content of honey is suspected to be the wind erosion of microfossils from the Tripoli Formation of Messinian age in nearby Sicily. In Poland, the source is similarly shown in the Polish Outer Carpathians, but with low diversity of silicoflagellata species. Atmospheric trajectory analysis confirmed the possibility of atmospheric transport of deflated grains in the harvesting period. Therefore, silicoflagellates could be used as indicators of the geographical origin of honeys collected in areas characterized by diatomite outcrops. It was demonstrated that the diatom content of honey can have both natural (wind) and artificial (filters) sources.

## Figures and Tables

**Table 1:** Silicoflagellates, diatoms, and cyanobacteria found in honey samples.

Name	Chloroplast	Major Group	Occurrence	Habitat	Sample Code	Country	Source
*Dictyocha fibula* Ehrenberg	no	Silicoflagellata	fossil	marine	FH29Af	Tunisia	floral
*Distephanopsis crux* (Ehrenberg)	no	Silicoflagellata	fossil	marine	FH29Af	Tunisia	floral
*Stephanocha speculum* (Ehrenberg)	no	Silicoflagellata	fossil	marine	FH25Af	Africa	floral
*Stephanocha speculum*	no	Silicoflagellata	fossil	marine	HP09Gr	Greece	honeydew, *Pinus*
*Stephanocha speculum*	no	Silicoflagellata	fossil	marine	FH33Po	Poland	floral, *Fagopyrum*
*Stephanocha speculum*	no	Silicoflagellata	fossil	marine	UK02Ro	Romania	unknown
*Stephanocha* cf. *speculum* (Ehrenberg)	no	Silicoflagellata	fossil	marine	FH28Af	Morocco	floral
*Stephanocha speculum speculum* (Ehrenberg)	no	Silicoflagellata	fossil	marine	FH29Af	Tunisia	floral
*Stephanocha speculum speculum f. notabilis* Locker & Martini	no	Silicoflagellata	fossil	marine	FH29Af	Tunisia	floral
*Achnanthes* sp.	no	Diatom	*	freshwater	UK05Cz	Czech Republic	unknown
*Achnanthes* sp.	living	Diatom	*	freshwater	UK05Cz	Czech Republic	unknown
*Achnanthidium* sp.	living	Diatom	*	freshwater	HH19It	Italy	honeydew
*Actiniscus pentasterias* Ehrenberg	no	dinoflagellates	fossil and actual	marine	FH29Af	Tunisia	floral
*Actinocyclus divisus* (Grunow) Hustedt	no	Diatom	fossil and actual	marine	FH29Af	Tunisia	floral
*Aulacodiscus* sp.	no	Diatom	*	marine	UK02Ro	Romania	unknown
*Aulacoseira distans* (Ehrenberg) Simonsen	no	Diatom	fossil and actual	freshwater	UK04Ge	Germany	unknown
*Aulacoseira distans* (Ehrenberg) Simonsen	no	Diatom	fossil and actual	freshwater	HC04It	Italy	floral, *Castanea*
*Aulacoseira distans* (Ehrenberg) Simonsen	no	Diatom	fossil and actual	freshwater	UK02Ro	Romania	unknown
*Aulacoseira* cf. *distans* (Ehrenberg) Simonsen	no	Diatom	fossil and actual	freshwater	FH33Po	Poland	floral, *Fagopyrum*
*Aulacoseira* sp.	no	Diatom	*	freshwater	UK02Ro	Romania	unknown
*Aulacoseira* sp.	no	Diatom	*	freshwater	HA05Gr	Greece	honeydew, *Abies*
*Chroococcus* sp.	living	Cyanobacteria	*	mainly freshwater	HA05Gr	Greece	honeydew, *Abies*
*Chroococcus* sp.	living	Cyanobacteria	*	mainly freshwater	UK07Sv	Switzerland	unknown
*Chroococcus* sp.	living	Cyanobacteria	*	mainly freshwater	HH29Sl	Slovakia	honeydew
*Chroococcus* sp.	living	Cyanobacteria	*	mainly freshwater	UK04Ge	Germany	unknown
*Chroococcus* sp.	living	Cyanobacteria	*	mainly freshwater	FH14Mx	Mexico	floral
*Chroococcus* sp.	living	Cyanobacteria	*	mainly freshwater	HH04It	Italy	honeydew
*Chroococcus* sp.	living	Cyanobacteria	*	mainly freshwater	HH04It	Italy	honeydew
*Chroococcus* sp.	living	Cyanobacteria	*	mainly freshwater	UK05Cz	Czech Republic	unknown
*Coscinodiscus* ? sp.	no	Diatom	fossil and actual	marine	FH33Po	Poland	floral, *Fagopyrum*
*Coscinodiscus* ? sp.	no	Diatom	fossil and actual	marine	FH29Af	Tunisia	floral
*Coscinodiscus marginatus* Ehrenberg	no	Diatom	fossil and actual	marine	FH29Af	Tunisia	floral
*Coscinodiscus* sp.	no	Diatom	fossil and actual	marine	UK02Ro	Romania	unknown
*Cyclostephanos dubius* Hustedt (Round)	no	Diatom	fossil and actual	freshwater	HA04Gr	Greece	honeydew, *Abies*
*Cyclostephanos dubius* Hustedt (Round)	no	Diatom	fossil and actual	freshwater	HA05Gr	Greece	honeydew, *Abies*
*Cyclotella* sp.	no	Diatom	fossil and actual	freshwater	HH31Hu	Hungary	honeydew
*Fragilaria* ? sp.	?	Diatom	?	freshwater	FH29Af	Tunisia	floral
*Fragilaria intermedia?* Grunow (Grunow)	living	Diatom	fossil? recent to actual	freshwater	UK05Cz	Czech Republic	unknown
*Fragilaria*? sp.	no	Diatom	*	freshwater	HH30Hu	Hungary	honeydew
*Hantschia amphioxys* (Ehrenberg) Grunow	no	Diatom	fossil and actual	freshwater	FH33Po	Poland	floral, *Fagopyrum*
*Hantzschia amphioxys* Ehrenberg (Grunow)	no	Diatom	fossil and actual	freshwater	HH31Hu	Hungary	honeydew
*Mastogloia* ? sp.	living	Diatom	*	freshwater/brackish	FH29Af	Tunisia	floral
*Nitzschia* ? sp.	no	Diatom	?	marine?	FH29Af	Tunisia	floral
*Nitzschia paleacea* Grunow in van Heurck	living	Diatom	*	freshwater	HH30Hu	Hungary	honeydew
*Nitzschia* sp.	living	Diatom	*	freshwater	FH20Cu	Cuba	floral
*Nitzschia* sp.	living	Diatom	*	freshwater	UK07Sv	Switzerland	unknown
*Nitzschia* sp.	living	Diatom	*	freshwater	FH31Hu	Hungary	floral, *Foeniculum*
*Nitzschia* sp.	living	Diatom	*	freshwater	FH31Hu	Hungary	floral, *Foeniculum*
*Oscillatoria* sp.	living	Cyanobacteria	*	mainly freshwater/marine	HA17Gr	Greece	honeydew, *Abies*
*Scenedesmus* sp.	living	green algae	*	freshwater	HA15Gr	Greece	honeydew, *Abies*
*Thalassionema nitzschioides* (Grunow) Mereschkowsky	no	Diatom	fossil and actual	marine	FH29Af	Tunisia	floral
centric sp.	no	Diatom	*	?	HH04It	Italy	honeydew
pennate sp.	living	Diatom	*	freshwater	HH08It	Italy	honeydew
pennate sp.	living	Diatom	*	freshwater	HA03Gr	Greece	honeydew, *Abies*

* for the living taxa not designated to species level, the geological record is not indicated.

## Data Availability

Data is contained within the article or supplementary material.

## Supplementary Materials

The following are available online at https://www.mdpi.com/2304-8158/10/2/421/s1, Supplement 1: The studied honey samples. Data are organized by collection code, botanical origin, type and location.

## References

[1] Soares S., Amaral J.S., Oliveira M.B.P., Mafra I. (2017). A comprehensive review on the main honey authentication issues: Production and origin. Compr. Rev. Food Sci. Food Saf..

[2] Mura-Mészáros A., Magyar D. (2017). Fungal honeydew elements as potential indicators of the botanical and geographical origin of honeys. Food Anal. Methods.

[3] Louveaux J., Maurizio A., Vorwohl G. (1978). Methods of melissopalynology. Bee World.

[4] Magyar D., Mura-Mészáros A., Grillenzoni F. (2016). Fungal diversity in floral and honeydew honeys. Acta Bot. Hung..

[5] Von Der Ohe W., Oddo L.P., Piana M.L., Morlot M., Martin P. (2004). Harmonized methods of melissopalynology. Apidologie.

[6] Beug H.J. (2004). Leitfaden der Pollenbestimmung für Mitteleuropa und Angrenzende Gebiete.

[7] Reille M. (1992). Pollen et Spores d’Europe et d’Afrique du Nord.

[8] Hamda S.H., Dhiab A.B., Galán C., Msallem M. (2017). Pollen spectrum in northern Tunis, Tunisia. Aerobiologia.

[9] Jilani B., Paul Schweitzer P., Larbi Khouja M., Zouaghi M., Ghrabi Z. (2008). Physicochemical properties and pollen spectra of honeys produced in Tunisia (Southwest Kef). Apiacta.

[10] El Gharbi B., Charpin H., Aubert J., Renard M., Mallea M., Soler M. (1976). Le calendrier pollinique de Tunis. Rev. Française D’allergologie D’immunologie Clin..

[11] Alekseyeva E.S., Bureyko A.L. (2000). Bee visitation, nectar productivity and pollen efficiency of common buckwheat. Fagopyrum.

[12] Zavada M.S., McGraw S.M., Miller M.A. (2007). The role of clothing fabrics as passive pollen collectors in the north-eastern United States. Grana.

[13] Mercuri A.M. (2015). Applied palynology as a trans-disciplinary science: The contribution of aerobiology data to forensic and palaeoenvironmental issues. Aerobiologia.

[14] Bagnold R.A. (1941). The Physics of Blown Sand and Desert Dunes.

[15] Xuan J. (2004). Turbulence factors for threshold velocity and emission rate of atmospheric mineral dust. Atmos. Environ..

[16] Leelőssy Á., Lagzi I., Mészáros R. (2017). Spatial and temporal pattern of pollutants dispersed in the atmosphere from the Budapest Chemical Works industrial site. Időjárás.

[17] Marticorena B., Bergametti G. (1995). Modeling the atmospheric dust cycle: 1. Design of a soil-derived dust emission scheme. J. Geophys. Res. Atmos..

[18] (2015). NOAA NCEP GDAS/FNL 0.25 Degree Global Tropospheric Analyses and Forecast Grids. Research Data Archive at the National Center for Atmospheric Research.

[19] Mészáros R., Leelőssy Á., Kovács T., Lagzi I. (2016). Predictability of the dispersion of Fukushima-derived radionuclides and their homogenization in the atmosphere. Sci. Rep..

[20] Leelőssy Á., Mészáros R., Kovács A., Lagzi I., Kovács T. (2017). Numerical simulations of atmospheric dispersion of iodine-131 by different models. PLoS ONE.

[21] McCartney K., Lipps J.H. (1993). Silicoflagellates. Fossil Prokaryotes and Protists.

[22] Dumitrica P. (2014). Double skeletons of silicoflagellates: Their reciprocal position and taxonomical and paleobiological values. Revue Micropaléontologie.

[23] Dumitrica P., Burns R.E., Andrews J.E., van der Lingen G.J., Churkin M., Galehouse J.S., Packham G.H., Davies T.A., Kennett J.P., Dumitrica P., Edwards A.R. (1973). Paleocene, late Oligocene and post-Oligocene silicoflagellates in southwestern Pacific sediments cored on DSDP Leg 21. Initial Reports of the Deep Sea Drilling Project, Vol. 21.

[24] Dumitrica P., Ryan W.B.F., Hsü K.J., Cita M.B., Dumitrica P., Lort J.M., Mayne W., Nesteroff W.D., Pautot G., Stradner H., Wezel F.C. (1972). Miocene and Quaternary silicoflagellates in sediments from Mediterranean Sea. Initial Reports of the Deep Sea Drilling Project, Vol. 13: 902–933.

[25] Rouchy J.M. (1982). La genèse des évaporites messiniennes de Méditerranée. Mémoires Muséum Natl. D’histoire Nat. Sér. C-Sci. Terre.

[26] Pestrea S., Blanc-Valleron M.M., Rouchy J.M. (2002). Les assemblages de diatomées des niveaux infra-gypseux du Messinien de Méditerranée (Espagne, Sicile, Chypre). Geodiversitas.

[27] Pellegrino L., Dela Pierre F., Natalicchio M., Carnavale G. (2018). The Messinian diatomite deposition in the Mediterranean region and its relationships to the global silica cycle. Earth-Sci. Rev..

[28] Krijgsman W., Hilgen F.J., Raffi I., Sierro F.J., Wilson D.S. (1999). Chronology, causes and progression of the Messinian salinity crisis. Nature.

[29] Deflandre G. (1967). La vie créatrice de roches. coll. Que sais-je? nr. 20.

[30] De Wever P., Cornée A. (2020). Roches à tout faire.

[31] Fenner J., Houben G., Kaufhold S., Lechner-Wiens H., Adams F., Baez J. (2012). Flying diatoms a key to the path and origin of a dust storm. Abstracts of the 22nd International Diatom Symposium.

[32] Schlichting H.E. (1971). A preliminary study of the algae and protozoa in sea foam. Bot. Mar..

[33] Geissler U., Gerloff J. (1965). Das Vorkommen von Diatomeen in menschlichen. Organen und in der Luft. Nova Hedwig..

[34] Folger D.W. (1970). Wind transport of land-derived mineral, biogenic, and industrial matter over the North Atlantic. Deep-Sea Res. Oceanogr. Abstr..

[35] Saint Martin S., Saint Martin J.P., Schmidt A.R., Girard V., Néraudeau D., Perrichot V. (2015). The intriguing marine diatom genus Corethron in Late Cretaceous amber from Vendée (France). Cretac. Res..

[36] Gregory P.H. (1961). The Microbiology of the Atmosphere.

[37] Ehrenberg G.G. (1844). Bericht über die zur Bekanntmachung geeigneten Verhandlungen. Königlich-Preuss. Akad. Der Wiss. Berl..

[38] Romero O.E., Lange C.B., Swap R., Wefer G. (1999). Eolian-transported freshwater diatoms and phytoliths across the equatorial Atlantic record: Temporal changes in Saharan dust transport patterns. J. Geophys. Res..

[39] Washington R., Todd M.C., Engelstaedter S., Mbainayel S., Mitchell F. (2006). Dust and the low-level circulation over the Bodele Depression. J. Geophys. Res..

[40] Sharma N.K., Rai A.K., Singh S., Brown Jr R.M. (2007). Airborne algae: Their present status and relevance 1. J. Phycol..

[41] Dumitrica P., Burns R.E., Andrews J.E., van der Lingen G.J., Churkin M., Galehouse J.S., Packham G.H., Davies T.A., Kennett J.P., Dumitrica P., Edwards A.R. (1973). Cenozoic endoskeletal dinoflagellates in Southwestern Pacific sediments cored during Leg 21 of the DSDP. Initial Reports of the Deep Sea Drilling Project, Vol. 21.

[42] Gläser G., Knippertz P., Heinold B. (2012). Orographic effects and evaporative cooling along a subtropical cold front: The case of the spectacular Saharan dust outbreak of March 2004. Mon. Weather Rev..

[43] Monteiro A., Fernandes A.P., Gama C., Borrego C., Tchepel O. (2015). Assessing the mineral dust from North Africa over Portugal region using BSC-DREAM8b model. Atmos. Pollut. Res..

[44] Alexandrowicz J. (2019). Personal communication.

[45] Figarska-Warchoł B., Stańczak G., Rembiś M., Toboła T. (2015). Diatomaceous rocks of the Jawornik deposit (the Polish Outer Carpathians): Petrophysical and petrographical evaluation. Geol. Geophys. Environ..

[46] Adeonipekun P.A. (2012). Palynology of honeycomb and a honey sample from an apiary in Lagos, Southwest Nigeria. Asian J. Plant Sci. Res..

[47] Buters J.T., Antunes C., Galveias A., Bergmann K.C., Thibaudon M., Galán C., Oteros J. (2018). Pollen and spore monitoring in the world. Clin. Transl. Allergy.

[48] Saint Martin S., Conesa G., Saint Martin J.P. (2003). Signification paléoécologique des assemblages de diatomées du Messinien dans le bassin de Melilla-Nador (Rif Nord-Oriental, Maroc). Rev. Micropaléontologie.

[49] El Ouahabi F.Z., Saint Martin S., Benmoussa A., Saint Martin J.P. (2007). Les assemblages de diatomées du bassin messinien de Boudinar (Maroc nord-oriental). Rev. Micropaléontologie.

[50] Stanimirova I., Üstün B., Cajka T., Riddelova K., Hajslova J., Buydens L.M.C., Walczak B. (2010). Tracing the geographical origin of honeys based on volatile compounds profiles assessment using pattern recognition techniques. Food Chem..

[51] Benmbarek M., Bonhomme C., Boussalem Z., Landbeck T. (2018). Mieux communiquer sur le miel, vers une nouvelle approche apiculteur-consommateur. Unpublished Memory MASTER 2 Management Administration des Entreprises.

[52] Borneck R., Gauthron R., Guiraute F., Horguelin P., Loveaux J., Pedelucq A. (1964). Les techniques de conditionnement et de commercialisation du miel au Canada et aux USA. Ann. L’abeille.

[53] Saint Martin J.-P., Métais G., Saint Martin S., Sen S. (2017). La diatomite du Coiron et son lagerstate. Géochronique.

[54] Genitsaris S., Kormas K.A., Moustaka-Gouni M. (2011). Airborne algae and cyanobacteria: Occurrence and related health effects. Front. Biosci..

[55] Tesson S.V., Skjøth C.A., Šantl-Temkiv T., Löndahl J. (2016). Airborne microalgae: Insights, opportunities, and challenges. Appl. Environ. Microbiol..

[56] Seijo C.M., Escuredo O., Fernández-González M. (2011). Fungal diversity in honeys from northwest Spain and their relationship to the ecological origin of the product. Grana.

[57] Harper M.A., McKay R.M., Smol J.P., Stoermer E.F. (2010). Diatoms as markers of atmospheric transport. The Diatoms.

