# Supplementary material for: The Occurrence of Skeletons of Silicoflagellata and Other Siliceous Bioparticles in Floral Honeys
Source: Foods. 2021 Feb 14;10(2):421. doi: 10.3390/foods10020421 (PMC7918607; doi:10.3390/foods10020421)
Supplement: Supplementary file 1 [file foods-10-00421-s001.pdf]

Supplement 1: The studied honey samples. Data are organized by collection code, botanical origin, type and location.

HH01Cr, unknown honeydew, Croatia; HH02It, unknown honeydew, Italy, Liguria; HH03It, unknown honeydew, Italy, Liguria; HH04It, unknown honeydew, Italy, Trentino Alto Adige; HH05It, unknown honeydew, Italy, Liguria; HA17Gr, *Abies alba* honeydew, Greece; HH07It, unknown honeydew, Italy, Lombardia; HH08It, unknown honeydew, Italy, Lazio; HH09It, unknown honeydew, Italy, Friuli Venezia Lucia; HH10It, unknown honeydew, Italy, Abruzzo; HH11It, unknown honeydew, Italy; HH12It, unknown honeydew, Italy, Piemonte; HH13It, unknown honeydew, Italy; HH14It, unknown honeydew, Italy, Liguria; HH15It, unknown honeydew, Italy, Liguria; HH17It, unknown honeydew, Italy, Toscana; HH18It, unknown honeydew, Italy, Toscana; HH19It, unknown honeydew, Italy, Toscana; HH20It, unknown honeydew, Italy, Toscana; HH21It, unknown honeydew, Italy, Liguria; HH22It, unknown honeydew, Italy, Liguria; HH23It, unknown honeydew, Italy, Liguria; HH24Hu, unknown honeydew, Hungary, Keszthely; FH28It, *Hedysarum coronarium* honey, Italy; HH26Hu, unknown honeydew, Hungary, Ózd; HH27Cr, unknown honeydew, Croatia; HH28It, unknown honeydew, Italy; HH29Sl, unknown honeydew, Slovakia; HA01It, *Abies alba* honeydew, Italy Liguria; HA02Gr, *Abies alba* honeydew, Greece; HA03Gr, *Abies alba* honeydew, Greece; HA04Gr, *Abies alba* honeydew, Greece; HA05Gr, *Abies alba* honeydew, Greece; HA06Gr, *Abies alba* honeydew, Greece; HA07It, *Abies alba* honeydew, Italy; HA08It, *Abies alba* honeydew, Italy; HA09Gr, *Abies alba* honeydew, Greece; HA10Gr, *Abies alba* honeydew, Greece; HA11It, *Abies alba* + *Picea excelsa* honeydew, Italy, Tuscan-Emilian Apennines; HA12Gr, *Abies alba* honeydew, Greece; HA13It, *Abies alba* honeydew, Italy; HA14Gr, *Abies alba* honeydew, Greece; HA15Gr, *Abies alba* honeydew, Greece; FH16Af, *Acacia* sp. honey, South Africa; FC01It, *Castanea sativa* honey, Italy (North); HC01It, *Castanea sativa* honeydew, Italy; HC02It, *Castanea sativa* honeydew, Italy; HC03It, *Castanea sativa* honeydew, Italy; HC04It, *Castanea sativa* honeydew, Italy; HC05It, *Castanea sativa* honeydew, Italy; HP01Gr, *Pinus brutia* honeydew, Greece; HP02Gr, *Pinus brutia* honeydew, Greece; HP03Gr, *Pinus brutia* honeydew, Greece; HP04Gr, *Pinus brutia*, honeydew, Greece; HP05Gr, *Pinus brutia* honeydew, Greece; HP06Gr, *Pinus brutia* honeydew, Greece; HP07Gr, *Pinus brutia* honeydew, Greece; HP08Gr, *Pinus* sp. honeydew, Greece; HP09Gr, *Pinus* sp. honeydew, Greece; FH01Sp, multifloral honey, Spain (Nortwest); FH02It, *Rhododendron* sp. honey, Italy, Alps; FH03Pt, *Rosmarinus officinale* honey, Portugal; FH04Sp, *Rubus* sp. honey, Spain (Northwest); FH05It, *Taraxacum officinale* honey, Italy, Piemonte; FH06It, *Tilia* sp. honey, Italy (North); FH07Af, unknown floral honey, South Africa; FH11Uz, unknown floral honey, New Zealand, Pohutukawa; FH12Af, unknown floral honey, Tansania; FH13Pt, unknown floral honey, Portugal; FH14Mx, unknown floral honey, Mexico; FH15It, *Citrus* sp. honey, Italy, Sicily; FH16Sp, *Eucalyptus* sp. honey, Spain, Coastal areas (North); FH17It, *Helianthus annuus* honey, Italy (Middle); FH18Sp, *Citrus* sp. honey, Spain; FH19Hu, *Asclepias syriaca* honey, Hungary, Szentkirály; FH20Cu, unknown floral honey, Cuba; FH21Af, unknown floral honey, Egypt; FH22Af, unknown floral honey, Tansania; FH23Af, *Citrus* sp. honey, South Africa; FH24Af, unknown floral honey, South Africa; FH25Af, unknown floral honey, Africa; FH26Af, unknown floral honey, South Africa; FH27Af, unknown floral honey, South Africa; FH28Af,

floral honey, Temara, Morocco; FH29Af, unknown floral honey, Tunisia; HH25Hu, unknown floral honey, Hungary, Solymár; HH30Sl, unknown honeydew, Slovakia; HH30Hu, unknown honeydew, Hungary Jász-Nagykun Szolnok County; HH31Hu, unknown honeydew, Hungary; HH31Po, unknown honeydew, Poland; HH31Bg, unknown honeydew, Bulgaria; UK01Ge, unknown, Germany, Altbulach; UK02Ro, unknown, Romania, Odorheiu Secuiesc/Székelyudvarhely; UK03Fr, unknown, France, Domaine St. Georges; UK04Ge, unknown, Germany, Altensteig-Überberg; HH29Hu, unknown honeydew, Hungary, Sárkeszi; UK05Cz, unknown, Czech Republic; UK06Cz, unknown, Czech Republic; UK07Sv, unknown, Switzerland, Les-ponts-de-Martel; FH30Hu, *Phacelia tanacetifolia* honey, Hungary, Oroszlány; FH31Hu, *Foeniculum vulgare* honey, Hungary, Sárkeszi; FH33Po, *Fagopyrum esculentum* honey, Stróze, Poland; FH34Po, *Fagopyrum esculentum* honey, Królów, Poland; FH35Po, *Fagopyrum esculentum* honey, Lipowy, Poland; FH36Po, *Fagopyrum esculentum* honey, Wiśniowa, Poland; FH37Po, *Robinia pseudo-acacia* honey, Stróze, Poland; HH32Po, honeydew honey, Pogorzany, Poland; HC06Po, *Castanea sativa* + honeydew honey, Poland.
